# SARS-CoV-2 and the Nucleus

**DOI:** 10.7150/ijbs.72482

**Published:** 2022-07-11

**Authors:** Mengqi Chen, Yue Ma, Wakam Chang

**Affiliations:** Department of Biomedical Sciences, Faculty of Health Sciences, University of Macau, Taipa, Macau, China

**Keywords:** SARS-CoV-2, nuclear transport, innate immunity, epigenetics, cilia, DNA damage

## Abstract

The ongoing COVID-19 pandemic is caused by an RNA virus, SARS-CoV-2. The genome of SARS-CoV-2 lacks a nuclear phase in its life cycle and is replicated in the cytoplasm. However, interfering with nuclear trafficking using pharmacological inhibitors greatly reduces virus infection and virus replication of other coronaviruses is blocked in enucleated cells, suggesting a critical role of the nucleus in virus infection. Here, we summarize the alternations of nuclear pathways caused by SARS-CoV-2, including nuclear translocation pathways, innate immune responses, mRNA metabolism, epigenetic mechanisms, DNA damage response, cytoskeleton regulation, and nuclear rupture. We consider how these alternations contribute to virus replication and discuss therapeutic treatments that target these pathways, focusing on small molecule drugs that are being used in clinical studies.

## Introduction

The ongoing coronavirus disease 2019 (COVID-19) is one of the deadliest infectious diseases in history. To date, it has caused more than 536 million confirmed cases and over 6.31 million deaths (*Weekly epidemiological update on COVID-19*, retrieved from https://www.who.int on 24 June 2022). COVID-19 is caused by severe acute respiratory syndrome coronavirus 2 (SARS-CoV-2), a member of the *Coronaviridae* family of enveloped RNA viruses 1. The *Coronaviridae* family includes the *Letovirinae* and *Orthocoronavirinae* subfamilies and members of the latter are commonly known as coronaviruses 2. The coronaviruses are categorized into four genera: *Alphacoronavirus*, *Betacoronavirus*, *Gammacoronavirus*, and Deltacoronavirus 2. SARS-CoV-2 belongs to the genus *Betacoronavirus*. Betacoronavirus can be further divided into four lineages, or subgroups 3,4. Common cold-causing HKU1 and OC43 belong to subgroup A, SARS-CoV and SARS-CoV-2 belong to subgroup B, and MERS-CoV is a member of subgroup C. All beta-coronaviruses are enveloped with a lipid bilayer that contains transmembrane proteins: membrane (M), envelope (E), and spike (S) structural proteins 5-7. The M and E proteins are essential for virion assembly and budding and determine the shape and size of the virus particles. The S protein forms surface projections and mediates viral entry into the host cells. It has been the center for anti-viral research and the major target for vaccine and therapeutic development. Inside of the envelope is the viral genome, a 26-32 kilobase (29.8-29.9 kilobase for SARS-CoV-2) positive-sense single-stranded RNA. The RNA genome is organized and protected by the nucleocapsid (N) structural protein. More than two-thirds of the SARS-CoV-2 genome encodes 16 nonstructural proteins (Nsp1-Nsp16), while the rest encodes S, E, M, N, and other open reading frames (ORFs) 6,7.

The binding of the S protein to its receptor, angiotensin-converting enzyme 2 (ACE2) for SARS-CoV-2, triggers membrane fusion and the viral genome is released into the cytosol. S protein contains an RGD tripeptide which can facilitate its binding to multiple integrins and viral entry into ACE2-negative cells 8-11. The virus can also enter monocytes using an antibody-Fcγ receptor-mediated mechanism 12. In the cytosol, the viral genome directs the expression of viral proteins to hijack cellular machinery 13,14. These include the transcription and translation machinery that are necessary for viral production and the innate immune responses that fight against the virus. In addition, the viruses also hijack cellular pathways to reduce cellular metabolism or repurpose them to optimize viral production. The exact pathways that are taken over vary greatly among viruses, and heavily depend on their life cycle. One of the most important factors contributing to pathway alternation is the site of replication, especially if the virus genome is replicated in the cytoplasm or in the nucleus 15. For viruses that are replicated in the nucleus, they must hijack nuclear import machinery to allow the viral genome to enter the nucleus and nuclear export machinery to transport the viral ribonucleoproteins out of the nucleus. Viruses that are replicated in the cytoplasm, including all positive-sense RNA viruses of eukaryotes, encode their own DNA or RNA polymerase, allowing the synthesis of their genomes outside of the nucleus.

Although coronavirus genome replication and transcription occur in the cytoplasm, the nucleus is important for their replication 16,17. A number of the viral proteins contain nuclear localization signals (NLSs) and/or display nuclear localization 18. Some also contain nuclear exporting signals 18. Several non-structural proteins were shown to alter the nuclear import and export functions to impair the translocation of transcription factors involved in immune responses 18. Here, we focus on SARS-CoV-2 and discuss the relationships between this coronavirus and the nucleus and their therapeutic implications.

## The nucleus and nuclear transportation

The life cycle of coronavirus doesn't show clear dependency on the nucleus, but virus clearance is accelerated by blocking nuclear entry and virus infection is reduced by inhibiting nuclear export 15,19,20, suggesting a role of the nucleus in virus replication. Virus replication was also shown to be greatly reduced in enucleated cells. The avian infectious bronchitis virus, a gamma-coronavirus, cannot replicate in enucleated cells 16. The murine hepatitis virus, a beta-coronavirus, can replicate in enucleated cells, but viral production is greatly decreased (down to 0.6 - 15% of control nucleated cells, dependent on the virus strains) 17. Replication of SARS-CoV-2 in enucleated cells has not been tested, but the above results suggest that the nucleus may similarly contribute to SARS-CoV-2 viral production.

The nucleus hosts almost all the cell's genomic material (except for mitochondrial DNAs) and contains nucleolus and a number of nuclear bodies involved in RNA synthesis, RNA processing, and ribosome assembly. It is the site of gene regulation, as transcription factors must enter the nucleus to activate or inhibit gene expression. The nucleus is bounded by the nuclear envelope consisting of the outer nuclear membrane (ONM), the inner nuclear membrane (INM), and the nuclear lamina. Between the ONM and the INM is the nuclear lumen, or the perinuclear space. Because the ONM is continuous with the endoplasmic reticulum (ER), the nuclear lumen is connected to the ER lumen. The INM and ONM are also continuous, but they contain different sets of proteins 21. Unanchored proteins diffuse freely on the planes of INM and ONM, but diffusion between these two layers is tightly regulated. The INM and ONM are connected by two types of protein complexes. First, they are connected by the linker of nucleoskeleton and cytoskeleton (LINC) complexes consisting of nesprins on the ONM and SUN proteins on the INM 22,23. The KASH motif of nesprins and the SUN domain of SUN proteins interact in the lumen and bridge the ONM and the INM. The LINC complexes couple the nucleoskeleton (the nuclear lamina) to the cytoskeleton and play important functions in mechanical signaling and force transduction. Second, they are connected by the nuclear pore complexes (NPC) 24. In fact, the ONM and the INM are continuous at the NPCs 21,24. The NPCs are large multi-protein pores on the nuclear envelope 24. The pores are open to both the cytoplasm and the nucleoplasm and are the sole gateway of transportation across the nuclear envelope. The nuclear lamina is composed of lamins (lamin A, B1, B2, and C) 25. These intermediate filament proteins form a meshwork underneath the INM and provide structural support to the nucleus. The lamina also provides attaching points for the LINC complexes, interacts with the NPCs, and through anchoring chromatids, regulates gene expression 25,26.

Nuclear transportation relies on the NPC and the gradient of Ran (Ras-related nuclear protein) small GTPase. The NPCs are giant pore complexes with eightfold rotational symmetry 24,27. Mammalian NPCs are about 110 MDa and are composed of multiple copies of ~30 different nucleoporins (Nups) 24,27. The inner diameter of an NPC is about 50 nm. However, the nucleoporins within the central channels contain repeating sequences of phenylalanine and glycine (FG repeats) that form disordered projections into the lumen 24. These projections create a permeability barrier and exclude the passage of large proteins. In general, small proteins (<50 kDa) can pass the NPC by passive diffusion while larger proteins require the transport receptors 24. This size limitation is approximate because the barrier is not stiff and surface residues of the cargo proteins can greatly alter the kinetics of their NPC passage 28,29. It can also be affected by post-translational modification. For example, glycosylation was shown to facilitate nuclear entry of BSA and neuroD1 30,31. In addition, as shown for YAP, mechanical stress on the nuclear membrane can stretch the nuclear pore to increase nuclear import 32.

**Figure 1:**
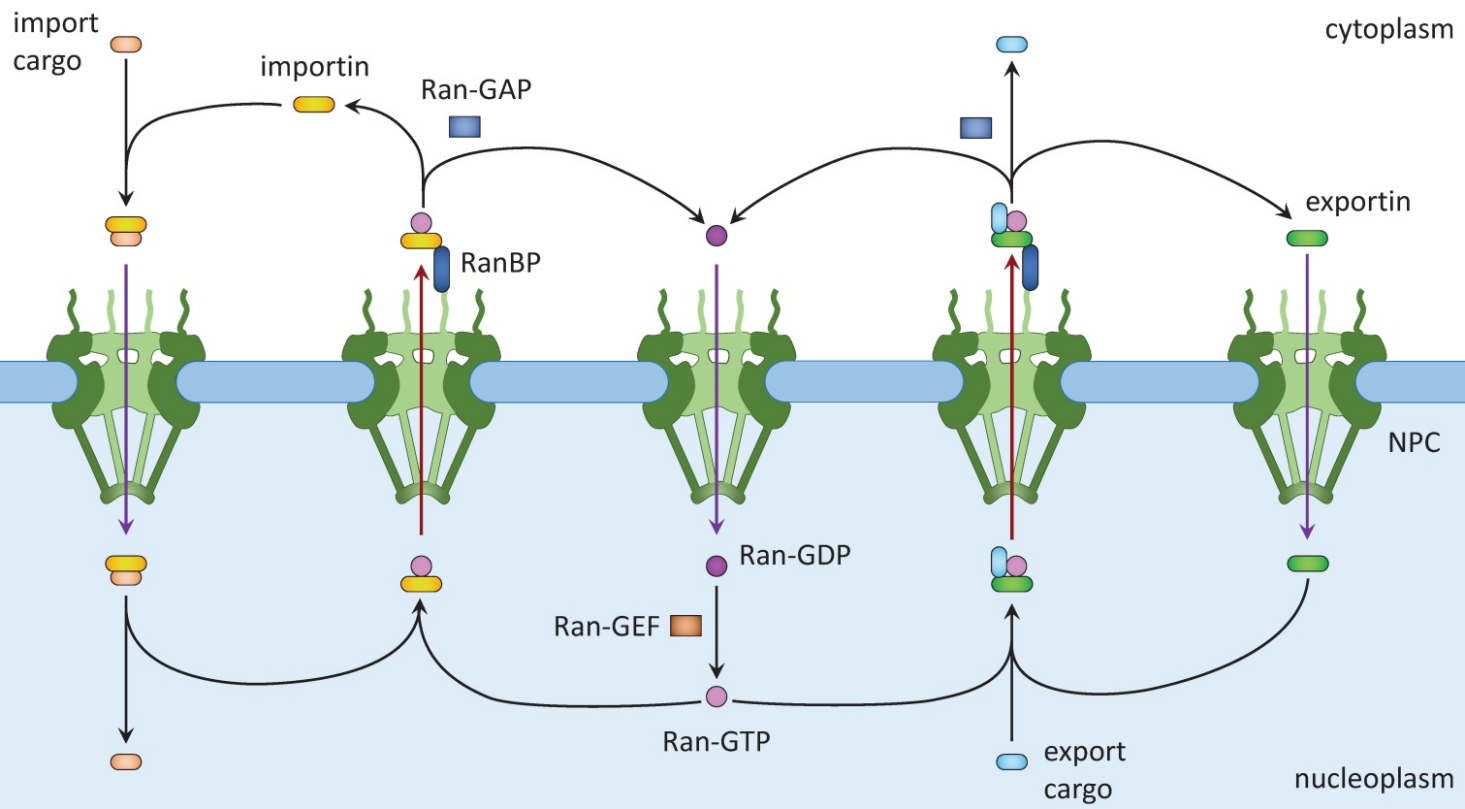
** Nuclear import and export. Left**: Cargo to be imported binds to cytoplasmic importin and enters the nucleus, where it is released from importin by Ran-GTP. The Ran-GTP/importin complex moves to the cytoplasm and binds RanBP. Ran-GAP activates the GTPase activity of Ran to convert Ran-GTP to Ran-GDP and release importin. Ran-GDP enters the nucleus and is recharged by a Ran-GEF. **Right**: Cargo to be exported binds to exportin and Ran-GTP in the nucleus. The complex moves to the cytoplasm where Ran-GTP is converted to Ran-GDP, leading to the disassembly of the complex. Both Ran-GDP and exportin then enter the nucleus for another round of nuclear export.

Active nuclear transport requires the Ran system. Ran is a member of the Ras family of small GTPases 15,18. Ran exists in two, GTP-bound and GDP-bound, forms. Ran binding proteins and Ran GTPase activating protein (GAP) in the cytoplasm facilitate the conversion of Ran-GTP into Ran-GDP, whereas RCC1, the nucleotide exchange factor (GEF) for Ran, converts Ran-GDP into Ran-GTP in the nucleus. Together, a Ran-GTP/Ran-GDP gradient is formed across the nuclear envelope. Nuclear import (Fig. 1, left) starts with the binding of NLS-containing cargos to importins, which allows them to shuttle between the cytoplasm and the nucleoplasm. Inside the nucleus, Ran-GTP binds to importin and releases the cargo, resulting in its nuclear accumulation. Ran-GTP and importin move together to the cytoplasm, in which Ran-GTP is converted to Ran-GDP and importin dissociates. Ran-GDP enters the nucleus and is converted to Ran-GTP there. Released importin and Ran-GTP can then participate in another round of nuclear importation. Nuclear export (Fig. 1, right) starts with nuclear export signal (NES)-mediated cargo (protein, ribonucleoprotein complex, or RNA) binding to Ran-GTP and an exportin. After passing through the NPC, the complex dissociates when Ran-GTP is hydrolyzed to Ran-GDP. Ran-GDP and exportin then diffuse into the nucleus for reuse.

## Viral replication and the nucleus

Upon membrane fusion, the coronavirus genomic RNA is uncoated and released into the cytosol. The released RNA first serves as an mRNA and is translated into polypeptides that are processed to form the replication and transcription complexes (RTC) 6,7. Some of the RTC subunits of SARS-CoV, namely Nsp3, Nsp4, and Nsp6, work together to remodel intracellular membranes, leading to the formation of convoluted membranes (CMs) and double-membrane vesicles (DMVs) 33. The SARS-CoV-2 Nsp3, 4, and 6 contain similar transmembrane domains as their SARS-CoV counterparts and are expected to function likewise in membrane rearrangement 34,35. Virus-induced membrane structures (CMs and DMVs) are collectively called the replication organelles, as they are believed to be the specialized sites of viral RNA synthesis and protect viral RNAs from being detected by the innate immunity 6. Cryotomographic studies revealed that the membrane of DMVs induced by the murine hepatitis coronavirus contains pore complexes that allow the export of synthesized RNAs to the cytosol 36. Viral infection also induces the formation of annulate lamellae, parallel stacks of ER-derived membranes. Annulate lamellae contain NPC proteins, suggesting a link to the nuclear envelope 37. The formation and functions of annulate lamellae are still poorly understood.

The genomic RNA contains a nested set of subgenomic mRNAs, which encode the M, E, S, and N structural proteins and additional accessory proteins 5. The N protein is expressed by cytoplasmic ribosomes and binds to replicated RNAs after they exit the replication organelles. The M, E, and S proteins are transmembrane proteins and, expectedly, are synthesized by rough ER-associated ribosomes and co-translationally translocated into the ER. Vesicles containing these proteins are transported to the ER-to-Golgi intermediate compartment (ERGIC). The ERGIC clusters, concentrated with viral proteins, then assemble around the newly formed N-encapsulated genomic RNAs, resulting in assembled virus particles in the lumen of secretory vesicles 5. The virus particles then go through the secretory pathway before being released via a lysosomal-dependent pathway 38.

The nucleus may contribute to virus production due to its connection to the replication organelles. Notably, the replication organelles are usually located in the perinuclear region of the cells. These organelles are interconnected and connect to the ER, so, in principle, material exchange can occur between the nuclear lumen and the intermembrane space of the DMVs. However, given that synthesized RNAs are protected by the inner membrane and enter the cytosol through the pore complex, it is unlikely that luminal factors can considerably affect virus replication. Similarly, transmembrane proteins synthesized in the ER can diffuse to the ONM. The E protein of SARS-CoV-2, when overexpressed alone, was indeed shown to localize to the nucleus (and the cytoplasm) 39. However, the functional significance of this localization is unknown.

The nucleus is also in close proximity to the ERGIC and the Golgi complexes. Two proteins involved in nuclear transportation, RanBP1 and importin-α, regulate the dynamics of the Golgi complexes 40-42. A pool of RanBP1 localized to the trans-Golgi network in neurons and mediates the nuclear export of proteins that regulate Golgi condensation 40. Importin-α directly interacts with GM130 on Golgi and functions in Golgi disassembly and spindle assembly during mitosis 41,42. Interestingly, importin-α may promote Golgi disassembly independent of Ran 41,43. The Golgi complexes are highly fragmented in SARS-CoV-2 infected cells 44. Whether this is due to altered nuclear transportation, however, has not been tested.

## Viral proteins in the nucleus

Supporting a role of the nucleus in viral production, several viral proteins were shown to contain NLS and/or NES and localize to the nucleus. The SARS-CoV N protein, which is 90% identical to SARS-CoV-2 N protein, contains multiple NLSs and a signal for nucleolar localization 18. Nucleolar localization was indeed observed for N protein of SARS-CoV 45. N protein of SARS-CoV-2, however, is mostly cytoplasmic 39. The S protein of SARS-CoV-2 was also shown to be present in the nucleus 39,46. In addition to the N and S proteins, many other viral proteins, when overexpressed, are detectable in the nucleus. These include E, ORF9a, Nsp1, Nsp3N, Nsp5 to Nsp7, Nsp9, Nsp10, and Nsp12 to Nsp16 39. Among them, only Nsp13 exhibits foci localization and enriches in the splicing compartment 39. Many of these proteins are small and probably enter the nucleus by passive diffusion.

Because viral assembly occurs in the cytoplasm, nuclear localization of some of the viral proteins may not be desirable and they need to be exported from the nucleus. Pharmacological inhibition of nuclear export leads to nuclear accumulation of viral proteins and significantly decreases viral infection 20. Several of the SARS-CoV-2 proteins, including N, S, M, E, Nsp9, Nsp12, and ORF3a, contain predicted NESs 18. These NESs may promote the nuclear export of these proteins or allow them to interact with exportins to hijack nuclear transportation (see below). The mechanism of nuclear export is best understood for SARS-CoV N protein. Phosphorylated N protein binds to adaptor protein 14-3-3 for nuclear export 47. SARS-CoV-2 N protein also binds to 14-3-3 in a phosphorylation-dependent manner 48.

## Targeting nuclear translocation of immune regulators

Pathogenic coronaviruses commonly interfere with the interferon (IFN) pathway but trigger an aberrant immune response by producing excessive cytokines and chemokines (cytokine storm) 14. IFNs are a family of antiviral cytokines produced and released by host cells upon detecting various pathogen-associated molecular patterns. There are three types of IFNs: Type I IFNs (including α and β) are anti-viral. The only type II IFN (γ) fights against both bacterial and virus infections. Whereas type III IFNs (λ) are produced after virus or fungal infections. All three types of IFNs are targets of coronaviruses. The expression of type I and III IFNs induced by SARS-CoV-2 is affected by many factors, like the tissue location, severity, and age 49. Type II IFN is also downregulated by SARS-CoV-2 50,51.

**Figure 2:**
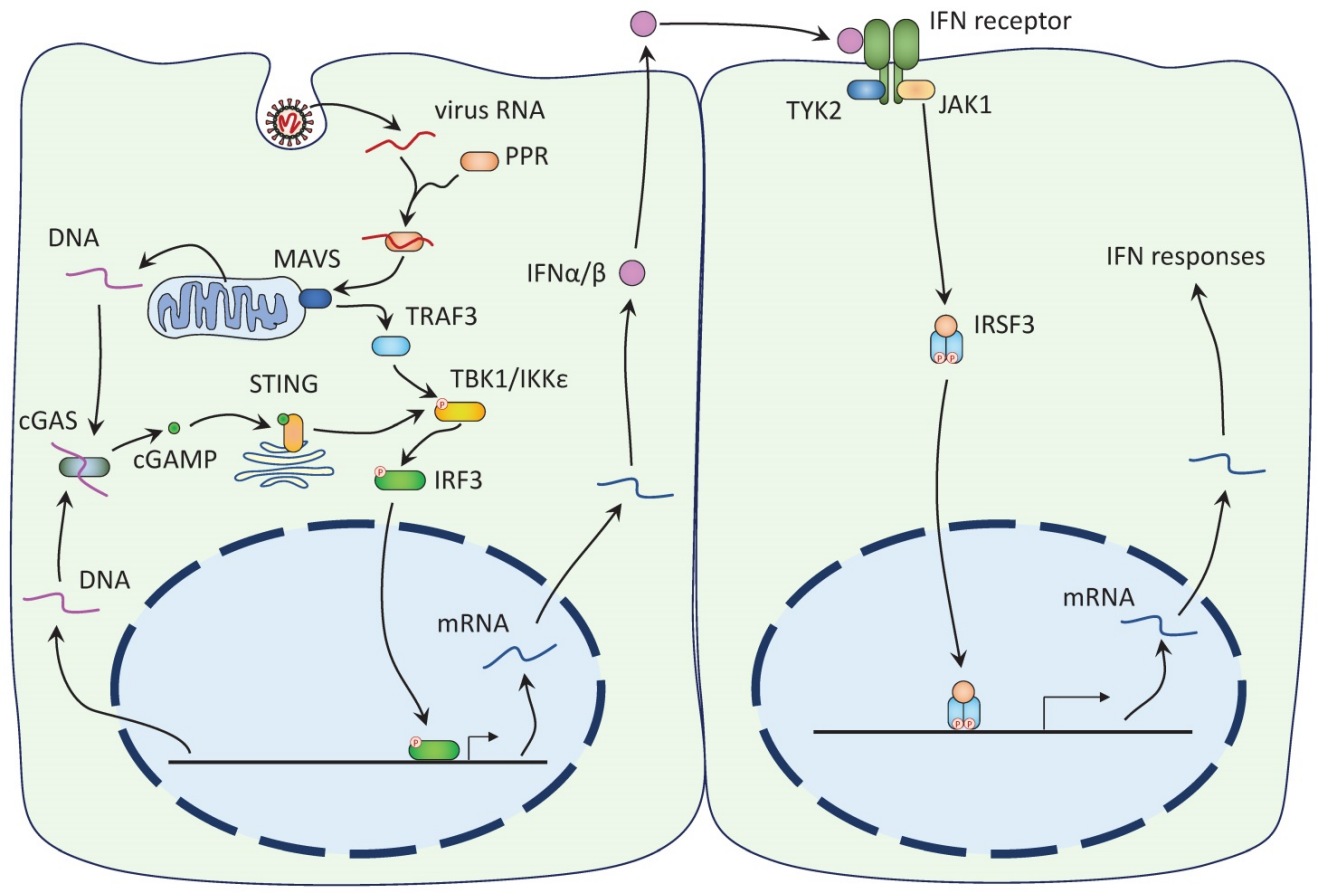
** SARS-Cov-2 induces the IFN-I response. Left**: 1) The viral RNA is recognized by host cell PPRs and together they activate MAVS on mitochondria. MAVS recruits TRAF3 to activate TBK1/IKKε, which in turn phosphorylates IRF3. IRF3 Phosphorylation leads to its nuclear translocation and the expression of the IFN-I genes. 2) DNA released from damaged mitochondria or ruptured nucleus is recognized by cGAS, leading to the synthesis of 2'3'-cGAMP. cGAMP binds to ER-localized STING and promotes its dimerization and translocation to the Golgi complex. STING activates TBK1 to induce IFN response. **Right**: Expressed IFNs are released and bind to the IFN receptors on the nearby cells. Receptor-associated JAK phosphorylates both STAT1 and STAT2, allowing them to dimerize and interact with IRF9 to form the ISFG3. The ISGF3 enters the nucleus and induces the expression of the IFN response genes.

The mechanisms of IFN antagonism have been extensively studied for type I IFNs 52,53. Type I IFN response begins with the recognition of virus RNA by host cell pattern recognition receptors (PPRs), like RIG-I and MDA5 (Figure 2). PPRs then bind to mitochondrial-associated adaptor MAVS and recruit and activate TRAF3. TRAF3 activates the interferon regulatory factors (IRF) kinases, TBK1 and IKKε, which phosphorylate IRF3 and IRF7. Phosphorylated IRF3/7 enter the nucleus to initiate the expression of the IFN genes. TBK1 and IKKε also phosphorylate IκB and facilitate the nuclear translocation of NF-κB, another important regulator of innate immune response. Expressed IFNs are released by the host cells and bind to the IFN receptors on the nearby cells to activate the Janus kinase (JAK)-STAT signaling cascade (Figure 2). Receptor-associated JAK phosphorylates and activates both STAT1 and STAT2, which, together with IRF9, form the IFN-stimulated gene factor 3 (ISGF3). The ISGF3 enters the nuclear with the help of importins α1/β1 and triggers the expression of antiviral genes 19.

SARS-CoV-2 antagonizes IFN with many, likely redundant, mechanisms targeting both the pre-IFN IRF3 pathway and the downstream JAK-STAT pathway (Table 1). Regulations on the mRNA level also exist (see below). In three studies, many of the viral proteins, including Nsp1, Nsp3, Nsp6, Nsp7, Nsp12, Nsp13, Nsp14, Nsp15, ORF3a, ORF6, ORF7a, ORF7b, and M, were shown to suppress IFN-α/β signaling, mostly by preventing the phosphorylation and subsequent nuclear entry of IRF3 54-56. In addition, Nsp5 was also reported to inhibit IFN response by targeting IRF3 57,58. Two viral proteins, Nsp2 and S, however, enhance IFN signaling 54. Several of these proteins, including Nsp1, Nsp3, Nsp6, Nsp13, ORF3a, ORF6, ORF7, and M, also inhibit nuclear translocation of STAT1/STAT2 54,55,59. Moreover, N protein was shown to inhibit the phosphorylation and nuclear translocation of STAT1/STAT2 60. It should be noted that these studies mostly relied on the ectopic expression of individual protein, and have identified overlapped but not identical sets of viral proteins 54-56.

The underlying mechanism of IFN downregulation is best understood for ORF6. SARS-CoV-2 ORF6 was shown to interact with importin α2 and inhibit importin-dependent nuclear translocation of both IRF3 and ISGF3 55. Interestingly, ORF6 of SARS-CoV also interacts with importin α2 and β1 and sequesters them on the ER and Golgi membrane 61. ORF6 additionally interacts with Nup98, an NPC component, and Rae1, a nuclear mRNA export factor, and interferes with nuclear trafficking, including STATs translocation 62-65. Inhibitors of importins are effective drugs for COVID-19, but the mechanism of action is still unclear 15,19,66.

The protease domain of Nsp3 (PL^Pro^) can downregulate IFN signaling by cleaving a ubiquitin-like protein, ISG15 59. Nsp3 prevents ISGylation of IRF3 by ISG15 and decreases both phosphorylation and nuclear translocation of IRF3 59. Directed cleavage of IRF3 by PL^Pro^ can also occur 58. Nsp5 also contains a protease domain and blocks nuclear translocation of phosphorylated IRF3, but this activity is independent of its protease domain 57. Nsp12 blocks the nuclear translocation of IRF3 without affecting its phosphorylation 67. Therefore, Nsp5 and Nsp12 may interact with the nuclear transport machinery. Both Nsp6 and Nsp13 bind to TBK1 55,68, but function differently to inhibit IRF3 phosphorylation. Nsp13 inhibits the phosphorylation of TBK1, whereas Nsp6 binds to phosphorylated TBK1 and inhibits its kinase activity 55,68.

In addition to the IFN responses, the virus also interferes with other anti-viral innate immune pathways. However, the inhibitions are not as tight as those of the IFN responses and whether nuclear entry of the transcription factors is altered by the virus is not well understood. Nevertheless, SARS-CoV-2 ORF9c interacts with proteins involved in NF-kB signaling 62 and NF-κB nuclear translocation is affected by the loss of Nup62 induced by Nsp9 expression 69, demonstrating that SARS-CoV-2 can alter NF-kB signaling through regulating its nuclear entry. The NF-κB pathways are critical for the cytokine storm 70. In the inactive state, the NF-κB heterodimer is sequestered in the cytoplasm by its inhibitor IκB. Inflammatory stimuli activate the IκB kinase, an enzyme complex consisting of IKKα kinase, IKKβ kinase, and a regulatory protein NEMO. Active IκB kinase phosphorylates IκB and causes its ubiquitinylation and proteasome degradation. Freed NF-κB then enters the nucleus to trigger the expression of inflammatory genes. The NF-κB pathways play dual roles in SARS-CoV-2 biology. On one hand, both Nsp5 and Nsp13 were shown to inhibit NF-κB 68,71. Nsp5 cleaves NEMO to reduce IFN signaling 71. On the other hand, Nsp5 was shown to activate NF-κB in another study 72. In addition, many viral proteins, including ORF3a, ORF7a, M, N, and S, activate NF-κB 11,73-76. Surprisingly, the NF-κB pathways may be essential for SARS-CoV-2, as its depletion results in a complete inhibition of virus replication 77.

Upstream of IFN and NF-κB, the cyclic GMP-AMP synthase-stimulator of interferon genes (cGAS-STING) pathway has a direct connection to the nucleus. Damage to the nuclear envelope or mitochondria releases DNA to the cytoplasm (Fig 2). Cytoplasmic DNAs are sensed by cGAS and induce the production of cyclic GMP-AMP, which binds to STING to activate TBK1 78. TBK1 then phosphorylates IRF3 to induce IFN production. Similar to the NF-κB pathways, cGAS-STING seems to play dual roles in COVID-19. ORF3a, ORF9b, N, and 3CL^Pro^ (protease domain of Nsp5) of SARS-CoV-2 were shown to inhibit STING 79,80 and pharmacological activation of cGAS-STING reduces virus infection 81,82. However, elevated cGAS-STING also contributes to the cytokine storm and inflammation 83,84.

## Targeting host mRNAs

SARS-CoV-2 cuts down cellular protein synthesis to inhibit immune responses and optimize viral replication. This is in part achieved by targeting host mRNAs, including inhibiting mRNA release after transcription, blocking nuclear trafficking of mRNAs, and accelerating degradation of mRNAs 85. SARS-CoV-2 induces the transcription of IFN mRNAs but most of these mRNAs are retained at the sites of transcription and degraded eventually in the nucleus 85. Escaped mRNAs can be transported to the cytoplasm, but global mRNA export is also inhibited by viral proteins. ORF6 interacts with Nup98 and Rae1 and impairs bidirectional nuclear transport, including host mRNA export 64,65. Nsp1, which suppresses host gene expression, also blocks mRNA export 86,87. The N-terminus of Nsp1 binds to NXF1, a subunit of the mRNA export receptor NXF1-NXT1. Nsp1 binding does not interfere with NXF1 binding to RNAs but impairs its interaction with nuclear export factors and the NPC 87. In the cytoplasm, exported host mRNAs face increased degradation mediated by Nsp1 85,86. Together, these studies demonstrated that SARS-CoV-2 targets multiple mRNA pathways to dampen the translation of host proteins.

## Coronavirus, cilia, and ciliary trafficking

Cilia are microtubule-dependent organelles projected from the cell body 88,89. Two types of cilia exist, the motile cilia that beat in coordinated waves to move extracellular material relative to the cell and the immotile primary cilia that act as cellular antennas and perform highly specialized sensory functions. Motile cilia play critical roles in the defense against airway infections 90. The respiratory tract is lined by epithelial cells that are mostly ciliated. Each ciliated epithelial cell has about two hundred cilia that beat in a coordinated fashion to propel the mucus layer and inhaled particles and pathogens out of the airways. SARS-CoV-2 preferentially targets ciliated cells and causes a rapid loss of the ciliated epithelium 90. Motile cilia in SARS-CoV-2 infected cells display structural abnormalities, both in shapes and sizes 90. The expression of Foxj1, a ciliogenesis regulator, is reduced by virus infection and may contribute to some of these cilia defects 90. Primary cilia are sensory organelles found in most non-blood cells in our bodies. With some exceptions, most cells possess one single primary cilium. The loss of smell and taste is a common symptom of SARS-CoV-2 infection and is related to cilia damage 91,92. ORF10 interacts with ZYG11B, an adapter protein of the CUL2 E3 ligase, and induces proteasomal degradation of proteins involved in ciliogenesis or cilium structure 93. Overexpression of ORF10 inhibits primary cilium assembly 93.

The machinery of nuclear trafficking has a surprising role in ciliogenesis and translocation of cilia proteins 88,89. At least ten Nups as well as importin-β1 and β2 are found to be present in cilia 89,94. The Nups localize to the base of the cilia and form a diffusion barrier to regulate protein translocation between the cilia and the cytoplasm 88,89. The structural organization of these Nups is poorly understood but is expected to share some features of the NPC. Similar to nuclear trafficking, ciliary transport depends on the FG repeats of the Nups and a ciliary Ran-GTP, cytoplasmic Ran-GDP gradient 88,89. Importins are also involved in ciliary transport. Importin-β1 interacts with CRB3-CLPI at the cilia and overexpression of an importin β1 mutant leads to strong cilia abnormalities 94. Importin β2 recognizes ciliary localization signals and mediates translocation of KIF17, an important component of the intraflagellar transport 95. Given that both SARS-CoV and SARS-CoV-2 ORF6s are reported to interact with and inhibit importins (α2 55 and β1 61), it is possible that ORF6 may affect ciliogenesis and cilia maintenance.

In a SARS‐CoV‐2-human protein interactomic study, Nsp13 is found to interact with 12 centrosomal components 62. The centrosome and the basal body of cilia are closely related microtubule-organizing centers and both contain centriole 62,96. Nsp6 of SARS‐CoV, when overexpressed alone, induces vesicle formation at the centrosome. Vesicle formation, however, does not occur when Nsp4 is coexpressed 33. The functional importance of these interactions is unknown.

## Epigenetic alternations by coronavirus

Epigenetic mechanisms, such as DNA/RNA methylation and histone modification, are important regulators of gene expression. Differences in epigenetic modifications contribute greatly to the susceptibility to SARS-CoV-2 and the prevalence of comorbidities 97,98. Epigenetic regulations of ACE2 expression and their effects on COVID-19 outcomes have been extensively studied and have great therapeutic potential 97-99.

Not surprisingly, SARS-CoV-2 also induces epigenetic changes to optimize viral infection and limit immunity. First, mRNA methylation is affected by virus infection. Epitranscriptomic studies using blood samples of COVID-19 patients and healthy controls have revealed significant differences in their N6-methylation of adenosine (m6A) modification profiles 100,101. METTL3, a subunit of the N6-adenosine-methyltransferase, is essential for m6A modification of SARS-CoV-2 RNAs and helps them to evade the RIG-I immune response 102-104. Depletion of METTL3 reduces virus infection in human lung fibroblasts but, surprisingly, enhances infection in human hepatocarcinoma cells 104,105. In infected cells, the expression of METTL3 is increased while the expression of FTO, a demethylase, is decreased 103,106. An increase in METTL3 expression relies on its interaction with the viral RNA-dependent RNA polymerase 106. Interestingly, while METTL3 and FTO are normally nuclear, cytoplasmic localization is observed in infected cells 106.

Second, DNA methylation is altered by SARS-CoV-2 and shows significant differences between COVID-19 patients and healthy controls. Profiles of DNA methylation can be used to calculate epigenetic ages and aging in COVID-19 patients was shown to be accelerated 107-109. Differentially methylated regions have been identified using blood, kidney, and heart samples, and many of these regions are close to genes involved in viral defense and interferon signaling 110,111.

Third, histone modification is altered by SARS-CoV-2. An interactomic study has identified direct interactions between viral proteins (E, Nsp5, Nsp8, and Nsp13) and epigenetic regulators 62. Inhibition of BRD2, a BET family reader of histone acetylation inhibition, reduces ACE2 expression and blocks SARS-CoV-2 112. Genome-wide studies of H3K4me3 and H3K27me3 modifications in peripheral blood mononuclear cells have demonstrated that genes related to immune response are upregulated after infection 113. NETosis, the formation of neutrophil extracellular traps (NET), is an antimicrobial cell death pathway 114. NETs contain modified chromatin and are characterized by the presence of citrullinated histone H3. Cell-free DNA and citrullinated histone H3 are increased in sera from patients with COVID-19 and correlate with the severity of the syndromes 115.

Lastly, chromatin organization contributes to viral susceptibility and is affected by the virus. Chromatin accessibility regulates the expression profiles of ACE2 and related genes and modulates differential virus entry across tissues 116. In a genome-wide CRISPR screen, the SWI/SNF chromatin remodeling complex was found to promote SARS-CoV-2 infection, suggesting a role of chromatin remodeling in virus life cycle 117. Single-cell transposase-accessible chromatin with sequencing experiments with peripheral blood mononuclear cells showed that COVID-19 induces significant chromatin remodeling 118,119. Changes in chromatin organization are enriched on genes of the inflammatory pathways and facilitate long-term adaptive immune responses 118,119. Drastic chromatin remodeling also occurs in olfactory sensory neurons and leads to significant downregulation of the olfactory receptor genes and, potentially, anosmia 117.

## Genetic alternations by coronavirus

One of the nuclear processes that is frequently triggered by virus infection is the DNA damage response, even for mRNA viruses 77,120,121. Proteins involved in DNA damage response are essential for virus infection as a small molecule inhibitor of the ATR kinase blocks SARS-CoV-2 replication 121. DNA damage response is quickly induced after SARS-CoV-2 infection but is swiftly suppressed in less than a day 77. This can be due to increased DNA damage caused by virus-induced nuclear rupture 81,122. Increased DNA damage and changes in the DNA damage response may lead to genomic changes or even genomic instability.

Besides DNA damage, SARS-CoV-2 was reported to affect two other aspects of the host genome. First, accelerated telomere shortening was found in infected Vero E6 cells and COVID-19 survivors 120,107. A telomere is the end region of a chromosome containing repetitive DNA sequences. Shortening of telomeres occurs during aging and increases the severity of COVID-19 123. Accelerated telomere shortening implies that aging is accelerated in COVID-19 patients. However, it should be noted that contradictory results, *i.e.* the lack of accelerated telomere shortening and accelerated aging, have also been reported 124. Second, virus genome and vaccine mRNA were reported to be reverse-transcribed and integrated into the host genome 125-127. These findings are highly debated due to the artificial expression of long interspersed nuclear element-1, a reverse transcriptase, and the concern of artifacts during library preparation 128-131.

## Other nuclear pathways affected by coronavirus

In addition to gating nuclear trafficking and altering epigenetic mechanisms, several nuclear pathways can also play roles in the virus life cycle or host cell anti-viral activities.

The nucleus may contribute to the virus life cycle indirectly through the cytoskeleton. All three classes of cytoskeletal elements (actin, intermediate filament, and microtubule) have been reported to involve in virus entry 132. The actin cytoskeleton plays several essential roles in viral entry. A recent report showed that myosin IIA directly interacts with the S protein of SARS-CoV-2 and facilitates virus infection 43. SARS-CoV-2 infection also stimulates casein kinase II and p38 MAP kinase pathways to induce actin polymerization 77. Nsp2 interacts with strumpellin, a component of the Wiskott-Aldrich syndrome protein and scar homology (WASH) complex, which regulates actin assembly and may contribute to virus egress 62. Intermediate filaments participate in virus replication and assembly 132. Vimentin intermediate filaments were shown to form a ''cage-like'' network around the DMVs and Withaferin A, a pharmacological inhibitor of vimentin, blocks viral replication 44. Lastly, the microtubule cytoskeleton is involved in ER-to-Golgi transportation of the virus particles 132. The nucleus senses mechanical forces and reorganizes the cytoskeleton through triggering mechanosignaling pathways, like the YAP/TAZ and serum response pathways 133. Lamin and INM protein emerin were shown to regulate actin dynamics 134. The LINC complexes couple the nucleus to the cytoskeleton and regulate Rho activity 135,136. The Rho family of small GTPases are master regulators of the cytoskeleton. SARS-CoV-2 may regulate Rho activity through Nsp7 binding to RhoA 62. S protein was also shown to activate RhoA 137. Alternatively, ORF3a interacts with SUN2 and may thus regulate the many functions of the LINC complexes, including Rho regulation 62. In addition, actin dynamics and the serum response can regulate NF-κB activity 138-140. MRTF, an activator of the serum response factor, binds to p65 in the nucleus and suppresses NF-κB activity 140. Similarly, lamin A/C deficiency reduces NF-κB-regulated transcription 138.

A recent study links the nucleus and the nuclear lamina to DNA-induced IFN response during SARS-CoV-2 infection 81. The nuclear lamina provides structural support to the nuclear envelope and protects it against mechanical stress. Defects in the nuclear lamina increase the frequency of nuclear envelope rupture, which exposes genomic DNA to cytoplasmic nucleases, damages DNA, and leads to genome instability 141. SARS-CoV-2 was shown to induce the cGAS-STING pathways because of the presence of cytoplasmic genomic DNAs, which are greatly increased by cell fusion 81,122. Interaction between the S protein and ACE2 can not only mediate virus entry, but also induce cell-cell fusion, resulting in the formation of syncytia - cells with multiple nuclei 142. Nuclei in the fused cells have lower levels of lamin A/C, the major contributor of the mechanical property of the nucleus, and have evident nuclear membrane blebs containing DNA 81. These blebs protrude from the nuclear lamina and rupture, leading to the release of DNA to the cytoplasm. Consistently, DNA damage foci, stained by γH2AX, are found to accumulate in infected cells with lower lamin A/C 81. diABZI, a potent STING activator, greatly reduces SARS-CoV-2 infection 81. These results suggest that nuclear integrity modulates host cell immune response during virus infection.

## Therapeutic implications

Small molecule drugs targeting the above pathways have therapeutic potential and some are being used in clinical studies. Epigenetic drugs have been reviewed and are not discussed here 143,144.

Because the IFN-I response is down-regulated by coronaviruses, many studies have been conducted to evaluate the effects of interferon treatments 145. In a study involving 11,330 subjects, the World Health Organization has found no benefit of IFN-β1 and recommends against using IFN-I for the treatment of COVID-19 146. The virus not only reduces IFN expression by blocking upstream signaling like the nuclear entry of IRF3, but also blocks the downstream JAK-STAT signaling by preventing nuclear translocation of ISGF3. Therefore, targeting nuclear trafficking may be more effective in fighting the virus and is a tempting alternative to IFN administration. The uses of inhibitors of nuclear transport in clinical studies have been previously summarized 15,18,19. Notably, Ivermectin, an FDA-approved importin α inhibitor, is included in almost 80 ongoing clinical trials for SARS-CoV-2 and has been shown to significantly improve viral clearance in several studies 15,19. Drugs against the nuclear export machinery are also shown to be beneficial for treating SARS-CoV-2. Selinexor and Verdinexor, drugs of the selective inhibitors of nuclear export (SINE) family, are FDA-approved and have been in clinical trials for treating COVID-19 18. Selinexor and Verdinexor target exportin-1 (also known as XPO1 and CRM1) and treatment of Selinexor inhibits the nuclear export of ORF3b, ORF9b, and N protein and induces anti-viral response 20. Selinexor treatment also leads to ACE2 accumulation in the nucleus 20.

Cytoplasmic DNAs can activate the cGAS-STING pathway and induce antiviral responses 81. Activators of the cGAS-STING pathway have been clinically tested for cancer therapy. One of which, diABZI, is an activator of STING and was shown to effectively block SARS-CoV-2 replication 81,82. However, STING activation is associated with SARS-CoV-2-induced inflammation and inhibition of cGAS-STING also exhibits beneficial effects 83,84,147. Compared to manipulating cGAS-STING, pharmacological inhibition of NF-κB may be more promising, as it can inhibit virus replication and dampen cytokine storm 70,148. Several NF-κB inhibitors have been used in clinical/preclinical trials 149.

DNA damage repair pathways play poorly understood roles in SARS-CoV-2 replication 121. In an antiviral drug screen, berzosertib, an inhibitor of the ATR kinase, exhibited potent anti-SARS-CoV-2 activity 121. Many pharmacological inhibitors of DNA damage repair have been identified and are used in clinical studies for cancer therapy 150. It is worth testing the effects of these drugs on SARS-CoV-2.

## Conclusion

We have summarized the evidence demonstrating the importance of the nucleus in SARS-CoV-2 infection. The nucleus plays multiple roles in the virus life cycle, but these roles are complicated and sometimes counteract each other. Some of the apparent incompatibilities are due to the differences between the *in vivo* and *in vitro* settings used in the studies. Or, it can be due to the differential effects of the virus on different cell types or different stages of infection. A better understanding of these roles of the nucleus requires more mechanistic studies.

Nuclear biology is understudied in COVID-19 research. The importance of the nucleus and its many interactions with the virus merit more studies in this area and promise fruitful results. Further works are expected to uncover new roles of the nucleus in SARS-CoV-2 infection and COVID-19 pathogenesis. They will also provide mechanical details of the SARs-CoV-2-nucleus interactions and fuel the discovery of new treatments.

## Figures and Tables

**Table 1:** Mechanisms of IFN down-regulation by SARS-CoV-2 proteins

	TBK1 phosphorylation	IRF3 phosphorylation	IRF3 translocation	STAT phosphorylation	ISGF3 translocation
Nsp1			Yes (55)	Yes (55)	Yes (55)
Nsp3		Yes (59)	Yes (59)		
Nsp5		No (57)	Yes (57)		
Nsp6	No (55)	Yes (55)	Yes (55)	Yes (55)	Yes (55)
Nsp7					
Nsp12		No (67)	Yes (67)		
Nsp13	Yes (55)	Yes (55)	Yes (55,56)	Yes (55)	Yes (55)
Nsp14			Yes (56)		
Nsp15			Yes (56)		
ORF3a				Yes (55)	Yes (55)
ORF6		No (54,55)	Yes (54-56)		Yes (55,63-65)
ORF7a				Yes (55)	Yes (55)
ORF7b				Yes (55)	Yes (55)
M				Yes (55)	Yes (55)
N				Yes (60)	Yes (60)

* Yes/No: whether the process (indicated by the column heading) is inhibited by the protein (indicated by the row label). The numbers in parentheses refer to the references.

## Abbreviations

COVID-19: Coronavirus disease 2019
SARS-CoV: Severe acute respiratory syndrome coronavirus
SARS-CoV-2: Severe acute respiratory syndrome coronavirus 2
M: membrane protein
E: envelope protein
S: spike protein
N: nucleocapsid
Nsp: nonstructural protein
ORF: open reading frame
ACE2: Angiotensin-converting enzyme 2
NLS: nuclear localization signals
NES: nuclear export signal
ONM: outer nuclear membrane
INM: inner nuclear membrane
ER: endoplasmic reticulum
ERGIC: ER-to-Golgi intermediate compartment
LINC: linker of nucleoskeleton and cytoskeleton
NPC: nuclear pore complex
Nup: nucleoporin
Ran: Ras-related nuclear protein
GAP: GTPase activating protein
GEF: nucleotide exchange factor
RTC: replication and transcription complexes
CM: convoluted membrane
DMV: double-membrane vesicle
IFN: Interferon
PPR: pattern recognition receptor
IRF: interferon regulatory factor
ISGF3: IFN-stimulated gene factor 3
cGAS: cyclic GMP-AMP synthase
STING: simulator of interferon genes
NF-κB: nuclear factor kappa B
NET: neutrophil extracellular traps
m6A: N6-methylation of adenosine
